# Distinct Cytokine Patterns Identify Acute and Convalescent Myocardial Involvement After Coronavirus Disease 2019: A Multicohort Biomarker Study

**DOI:** 10.1093/infdis/jiaf045

**Published:** 2025-02-19

**Authors:** David A Zidar, Grace A McComsey, Donald D Anthony, John McDaniel, Timothy A Chan, Sadeer G Al-Kindi

**Affiliations:** Departments of Pathology and Medicine, Case Western Reserve University School of Medicine; Department of Medicine, University Hospitals Cleveland Medical Center; Department of Pathology, Louis Stokes Cleveland Veterans Affairs Medical Center, Cleveland; Departments of Pathology and Medicine, Case Western Reserve University School of Medicine; Department of Medicine, University Hospitals Cleveland Medical Center; Departments of Pathology and Medicine, Case Western Reserve University School of Medicine; Department of Pathology, Louis Stokes Cleveland Veterans Affairs Medical Center, Cleveland; Department of Pathology, Louis Stokes Cleveland Veterans Affairs Medical Center, Cleveland; School of Health Sciences, Kent State University, Kent; Global Center for Immunotherapy and Immuno-Oncology, Cleveland Clinic Lerner College of Medicine, Cleveland, Ohio; Departments of Pathology and Medicine, Case Western Reserve University School of Medicine; Department of Medicine, University Hospitals Cleveland Medical Center

**Keywords:** interleukin 18, tumor necrosis factor, cardiopulmonary exercise testing, cardiac MRI, postacute sequelae of COVID-19

## Abstract

We sought to identify the immunobiologic underpinnings of cardiac involvement as a postacute sequela of coronavirus disease 2019 (COVID-19) by comparing acute and convalescent populations. For the latter, an integrated analysis of cytokine levels, cardiac magnetic resonance imaging, and cardiopulmonary exercise capacity was performed. Unlike acute cardiac injury, which was associated with heightened tumor necrosis factor alpha (TNF-α) but not interleukin 18 (IL-18), convalescent myocardial inflammation/edema correlated with IL-18 but not TNF-α. Thus, inflammation is not a monolith in relation to cardiac involvement in the setting of COVID-19. Instead, convalescent cardiac involvement may emerge from mechanisms distinct from acute injury, and appropriately targeted therapies may prevent postacute sequalae of COVID-19.

## Teaser

In this integrated analysis of immune activation, cardiac magnetic resonance imaging, and cardiopulmonary exercise testing, the immunologic correlates of acute versus chronic myocardial involvement differed. Cardiovascular resilience in the aftermath of COVID-19 therefore ultimately requires avoidance of multiple distinct time-dependent immunopathologic processes.

Severe acute respiratory syndrome coronavirus 2 (SARS-CoV-2) infection can lead to cardiac injury acutely, which has been associated with hyperinflammation and heightened mortality [1, 2]. Among recovered survivors, protracted cardiopulmonary disability is common and cardiac edema/inflammation (ie, T2 signal by cardiac magnetic resonance imaging [cMRI]) has been reported [3, 4]. However, the specific mechanisms that link immune activation/convalescence to cardiac injury and repair across the time course of coronavirus disease 2019 (COVID-19) survivorship has not been established. We sought to test the hypothesis that cardiac involvement in the postrecovery phase may stem from activation of pathways distinct and separable from those responsible for acute myocardial injury.

## METHODS

We conducted a comparative multicohort immune biomarker study of acutely hospitalized adults (cohort 1) and posthospitalized, convalescent adults (cohort 2) receiving care at University Hospitals Cleveland Medical Center. Cohort 1 (n = 37) included patients hospitalized for COVID-19 between 1 August 2020 and 30 July 2021 who had ≥1 troponin level measured during clinical care. Cohort 2 participants (n = 28) were also hospitalized during the first wave (23 March 2020 to 10 September 2021), but survived and were eligible regardless of protracted symptom burden. This cohort was studied ≥3 months removed from infection. Per research protocol, cohort 2 participants completed (1) the Duke Activity Score Index (DASI) questionnaire to assess symptom burden, (2) underwent cardiopulmonary exercise testing (CPET) to quantify cardiopulmonary exercise capacity, and (3) underwent cMRI (1.5T Siemens Aera).

Peripheral blood was drawn during acute hospitalization (cohort 1) or after recovery (cohort 2) and plasma cytokines were measured using the MesoScale Discovery platform (Rockville, Maryland). Immunologic predictors included tumor necrosis factor alpha (TNF-α), interleukin 8 (IL-8), interleukin 6 (IL-6), and interleukin 18 (IL-18) levels and primary outcomes were cardiac injury (peak troponin I ≥0.03 ng/mL) and T2-weighted signal intensity (continuous) in the acute and recovery cohorts, respectively. Convenience sample sizes were recruited and standard descriptive statistics, multivariable regression models, and principal component analysis (PCA) were used to describe relationships. Statistical Package for the Social Sciences (IBM) was used for analyses and Biorender was used for image creation. This study was approved by the institutional review board of University Hospitals of Cleveland Medical Center and written informed consent was required prior to patient participation.

## RESULTS

**Figure 1.**
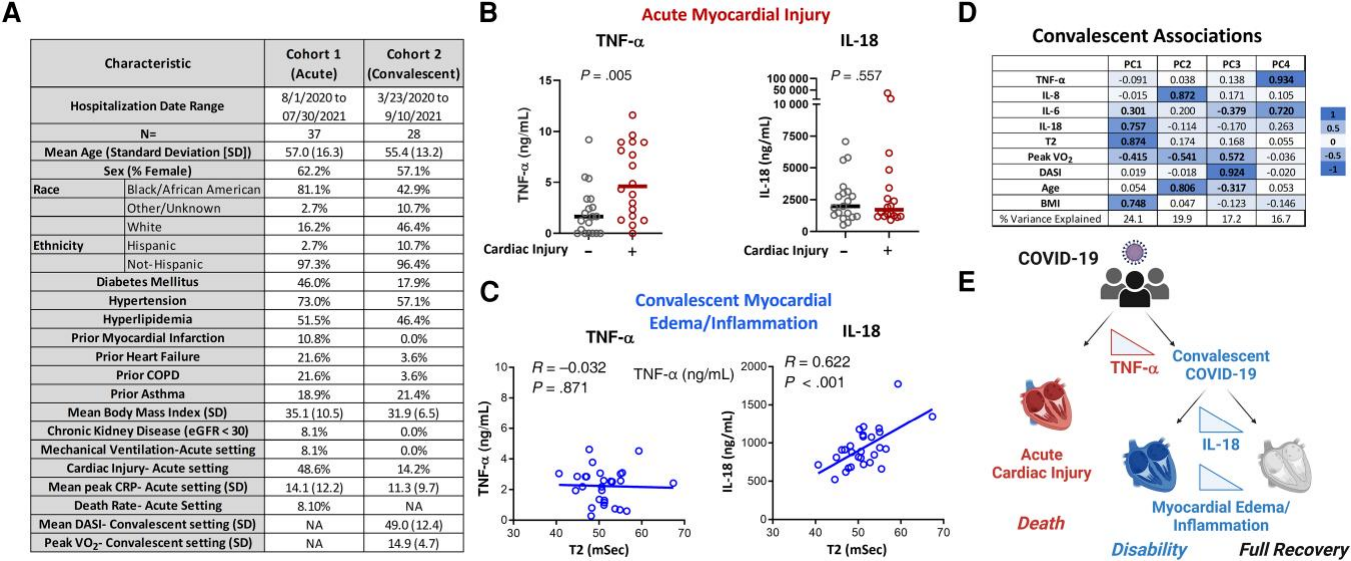
Distinct cytokine patterns identify acute and convalescent myocardial involvement after coronavirus disease 2019 (COVID-19). *A*, Clinical characteristics of cohorts 1 and 2 are shown. *B*, Plasma levels of tumor necrosis factor alpha and interleukin 18 are shown in cohort 1 participants, comparing those with versus without acute COVID-19 cardiac damage (independent *t* tests). *C*, Scatterplots and correlation analyses show cytokine levels in relation to myocardial T2-weighted signal intensity by cardiac magnetic resonance imaging during convalescence (cohort 2). *D*, The loadings (PC1–4) from a varimax-rotated principal component analysis of data from cohort 2 are shown as raw values and heatmap. *E*, A conceptual model is presented whereby multiple inflammatory pathways determine cardiac involvement and clinical sequelae after COVID-19. Abbreviations: BMI, body mass index; COPD, chronic obstructive pulmonary disease; COVID-19, coronavirus disease 2019; CRP, C-reactive protein; DASI, Duke Activity Score Index; eGFR, estimated glomerular filtration rate; IL, interleukin; NA, not applicable; PC, principal component; SD, standard deviation; TNF-α, tumor necrosis factor alpha.

In cohort 1, the mean age was 57.0 (standard deviation [SD], 16.3) years, 62.2% were female and 16.2% White, and 18 of 37 patients had cardiac injury (troponin I ≥0.03 ng/mL). Cohort 2 had a mean age of 55.0 (SD, 13.2) years, 57.1% were female and 46.4% White, and participants were analyzed an average of 224 (SD, 119) days after positive SARS-CoV-2 testing, at which time substantial disability was evident based upon patient-reported symptoms (mean DASI, 49.0 [SD, 12.4]) and objective CPET measures (mean maximal oxygen uptake [VO_2_ max], 14.9 [SD, 4.7]). Additional clinical characteristics for these cohorts are shown (Figure 1*A*).

To identify the immunologic pathways most relevant to acute cardiac injury, several cytokines were measured, comparing those with evidence of cardiac injury with those who were hospitalized but did not have elevated troponin levels. Myocardial injury in the acute setting was associated with elevated TNF-α levels (Figure 1*B*, left panel), and this relationship was independent of age, sex, racial/ethnic differences, and body mass index (adjusted odds ratio, 3.9 per SD of TNF-α [95% confidence interval {CI}, 1.1–14.2]; *P* = .04). IL-8 and IL-6 were less strongly related to acute cardiac injury (not shown) and IL-18 levels were similar in those with versus those without myocardial necrosis (Figure 1*B*, right panel).

In the convalescent cohort, cytokines were measured and compared to T2 signal intensity, a surrogate of myocardial edema and/or inflammation. T2 was not associated with TNF-α (Figure 1*C*, left panel), but strongly correlated with higher IL-18 levels (Figure 1*C*, right panel), an association independent of demographics and time since infection (standardized β = .51 [95% CI, .20–.81]; *P* = .002).

To characterize the correlates of cardiopulmonary disability (VO_2_ max) after recovery from COVID-19, we used PCA to identify patterns of shared variation among these biomarkers. This analysis (Figure 1*D*) suggests that multiple factors influence cardiopulmonary capacity after COVID-19 recovery. Principal component (PC) 1 indicated a high degree of shared variance involves systemic IL-18 levels, T2 cMRI signal intensity, body mass index, and reduced peak VO_2_. Diminished cardiopulmonary capacity was also partially related to heightened IL-8 levels in conjunction with aging without a strong connection to cMRI T2 signal intensity (PC2). Neither PC1 nor PC2 was associated with patient-reported symptoms, which instead were closely aligned with PC3. Variance in convalescent TNF-α levels was related to IL-6 variance but was not strongly associated with peak VO_2_ or with myocardial inflammation.

## DISCUSSION

Our findings are consistent with prior evidence that cardiac injury in the acute phase of COVID-19 is associated with heightened TNF-α, as previously described and consistent with its known deleterious effects on the endothelium and cardiomyocytes [1, 2]. New in this study is that TNF-α levels did not appear to correspond with myocardial edema/inflammation in recovery and were unrelated to symptom burden and cardiopulmonary fitness. Instead, systemic IL-18 was the strongest indicator of myocardial edema/inflammation by cMRI after recovery from COVID-19. This is particularly notable given the lack of association between IL-18 levels and acute myocardial injury.

Prior studies have raised the concern for protracted myocardial inflammation/edema after COVID-19 infection, but this is the first study to identify the systemic immune correlates thereof. A relationship between IL-18 and myocardial edema/inflammation is plausible, either as an epiphenomenon of protracted inflammasome activation and/or as a direct contributor. To our knowledge, IL-18 has not been previously reported to associate with T2 signal intensity or postacute sequalae of COVID-19 (PASC), but has been linked to metabolic syndrome and vaccine-induced myocarditis [5].

Conceptually, these data suggest that a high level of specificity governs the relationship between cardiac involvement and the various inflammatory pathways triggered during COVID-19. Our data suggest that acute cardiac injury and protracted myocardial inflammation/edema are best viewed as potentially separable consequences. For instance, convalescence from TNF-α alone did not ensure avoidance of myocardial edema/inflammation or a return to optimal cardiopulmonary capacity. This has important implications for the development of precise therapeutic strategies and could also explain the observed lack of relationship between acute disease severity and cMRI manifestations [3, 4]. The PCA is also consistent with specificity between certain clinical features (eg, age, adiposity) and distinct inflammatory pathways in that IL-18 was closely related to adiposity but not age (PC1) whereas the opposite was true for IL-8 (PC2). And while each component contributed to diminished exertional capacity, only the former associated with myocardial inflammation/edema. These data therefore provide novel insight into the complexity of post-COVID-19 recovery, support a model whereby acute and convalescent cardiac involvement after COVID-19 arise from distinct mechanisms (Figure 1*E*), and point to new therapeutic strategies for PASC.

This study was limited by modest sample sizes, and the long-term implications of cMRI findings in this setting remain unknown. Whether the observed linkages between IL-18 and T2 intensity extend to other clinical settings, including healthy aging, will require further study. Studies to validate this relationship, identify the source of IL-18, and determine whether it is cause, consequence, or epiphenomenon of myocardial pathology are needed to extend our mechanistic understanding of recovery from SARS-CoV-2 and other viral infections.

## Note

***Financial support*.** This work was supported by the National Cancer Institute (grant number U01CA260513 to D. A. Z. and T. A. C.); the United States Veterans Administration (grant number COVID19-8900-05 to D. A. Z.); and the Clinical and Translational Science Collaborative of Cleveland (grant number UM1TR002548 to G. A. M.), from the National Center for Advancing Translational Sciences.
